# Supplementary material for: SenCat: Cataloging human cell senescence through multiomic profiling of multiple senescent primary cell types
Source: bioRxiv. 2026 Feb 7:2026.02.05.703986. Preprint. [Version 1] doi: 10.64898/2026.02.05.703986 (PMC13004134; doi:10.64898/2026.02.05.703986)
Supplement: Supplement 2 [file media-2.pdf]

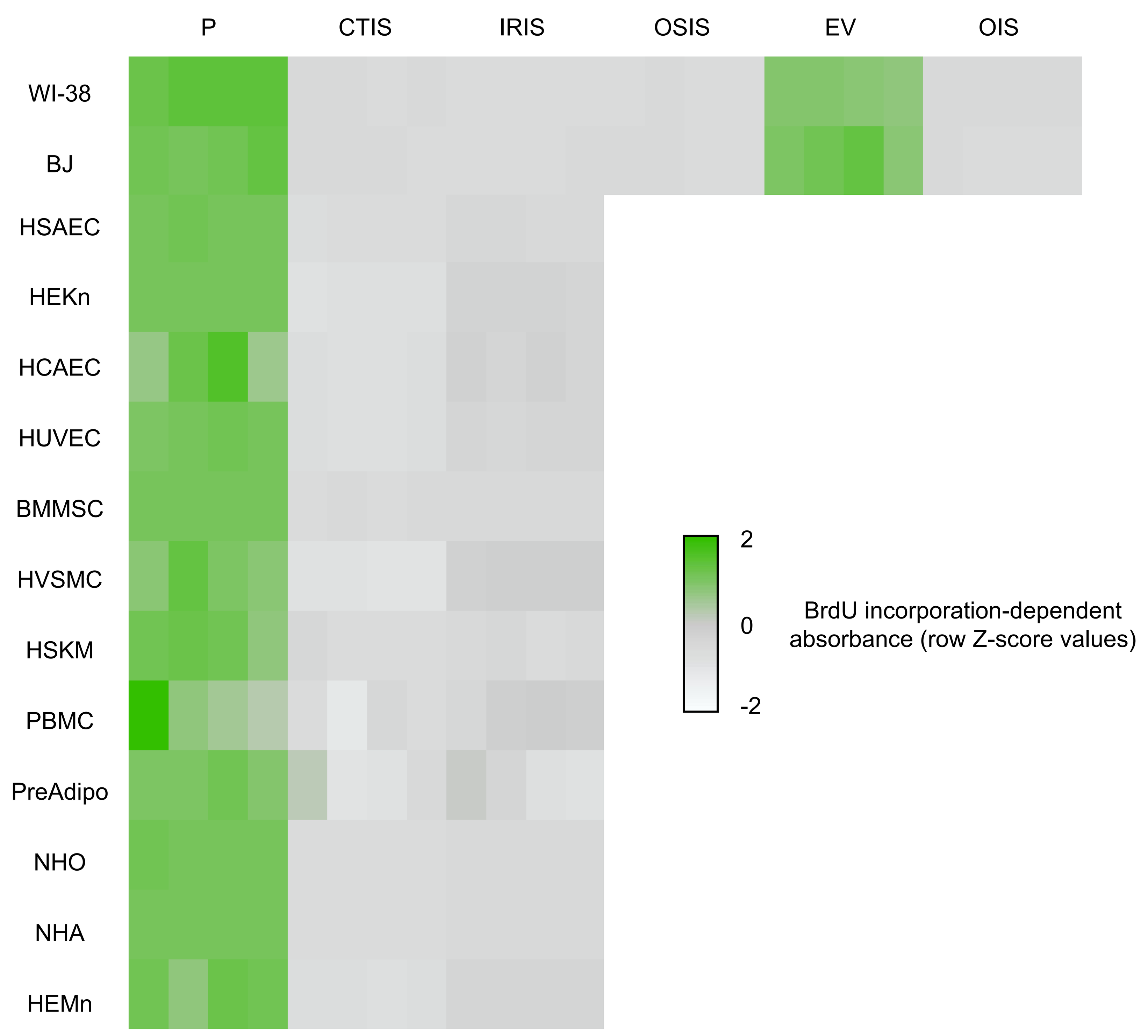
P
CTIS
IRIS
OSIS
EV
OIS
WI-38
BJ
HSAEC
HEKn
HCAEC
HUVEC
BMMSC
HVSMC
HSKM
PBMC
PreAdipo
NHO
NHA
HEMn
2
0
-2
BrdU incorporation-dependent absorbance (row Z-score values)

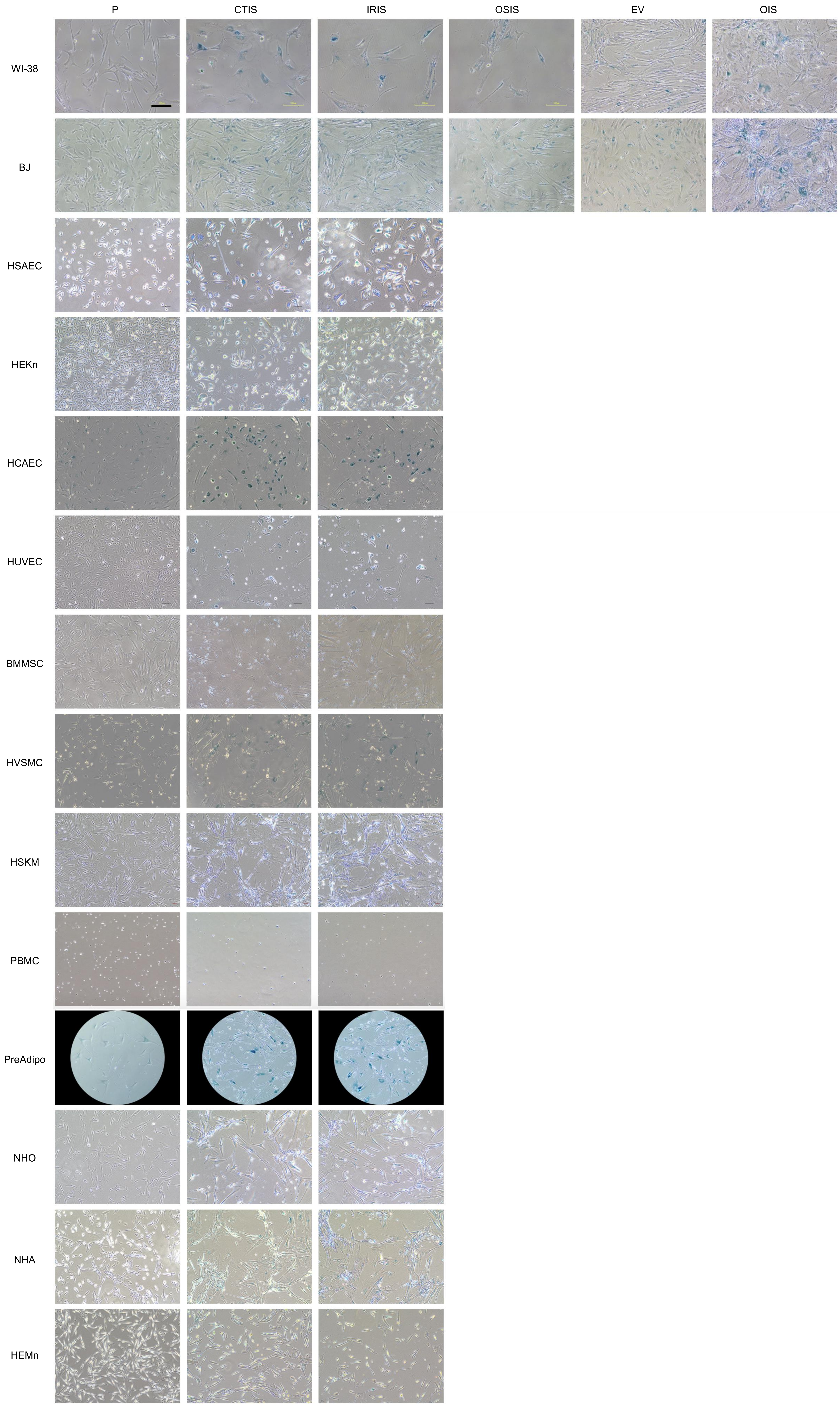
P
CTIS
IRIS
OSIS
EV
OIS
WI-38
BJ
HSAEC
HEKn
HCAEC
HUVEC
BMMSC
HVSMC
HSKM
PBMC
PreAdipo
NHO
NHA
HEMn

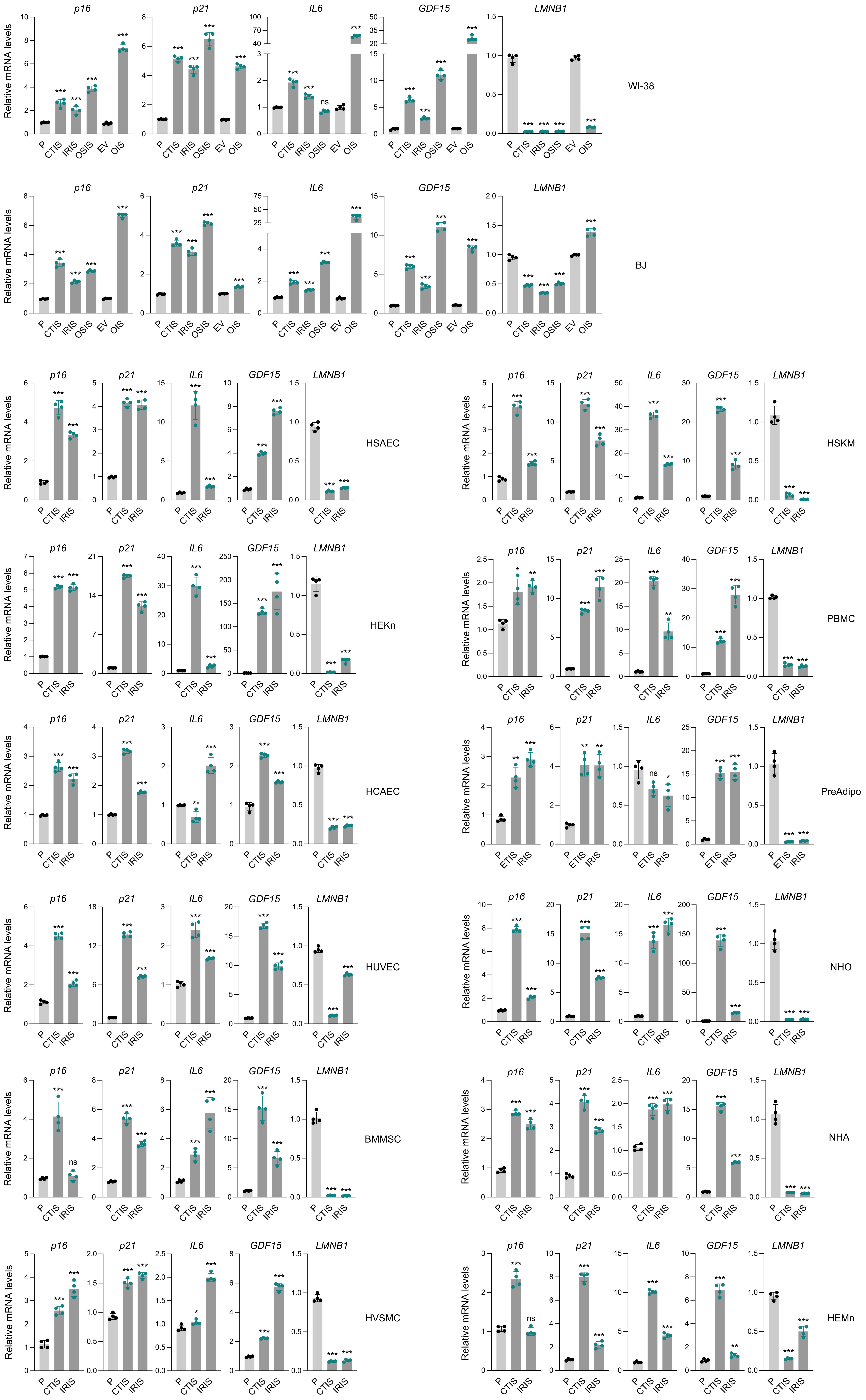
p16
p21
IL6
GDF15
LMNB1
Relative mRNA levels
P
CTIS
IRIS
OSIS
EV
OIS
ETIS
ns
WI-38
BJ
HSAEC
HSKM
HEKn
PBMC
HCAEC
PreAdipo
HUVEC
NHO
BMMSC
NHA
HVSMC
HEMn

A

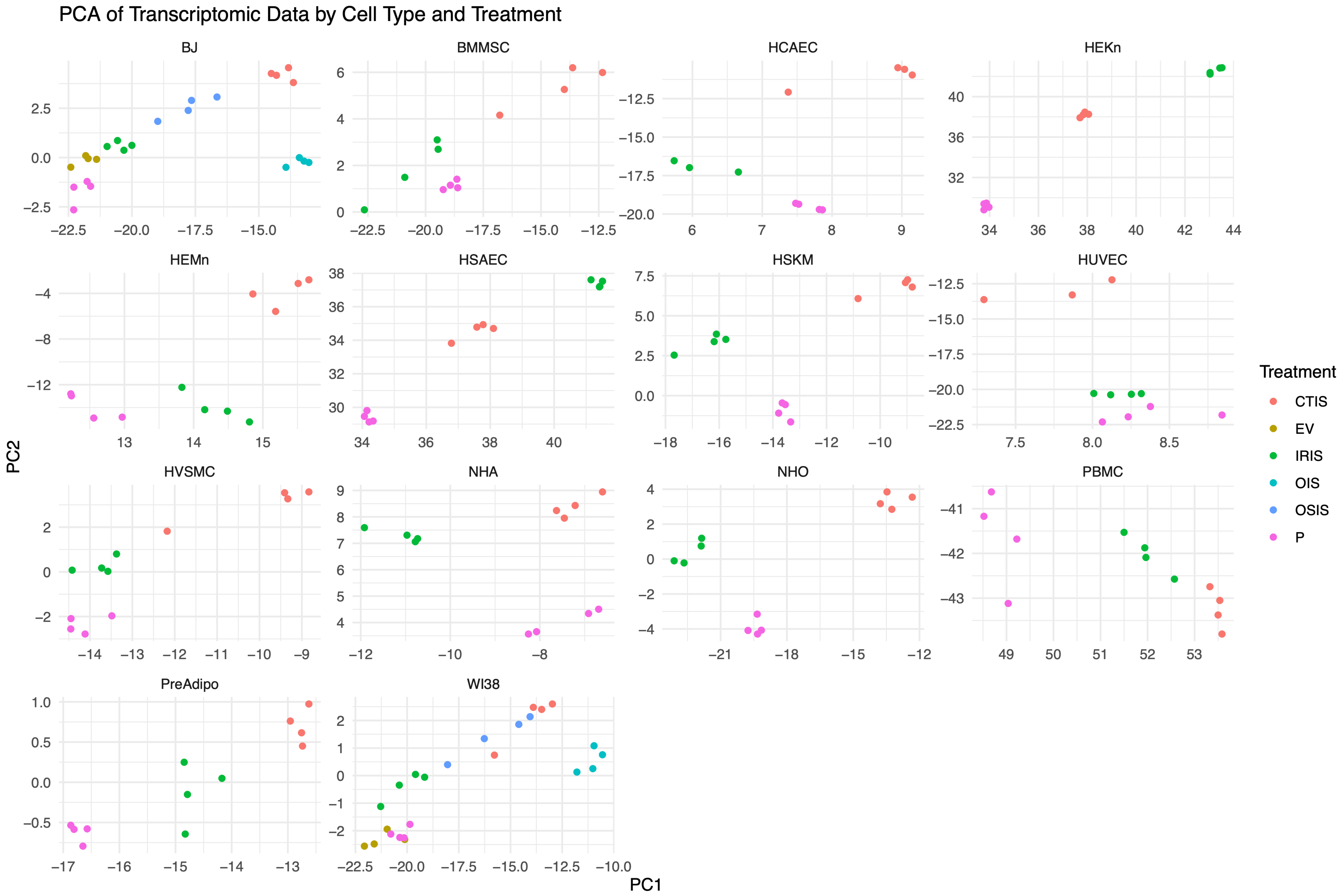
PCA of Transcriptomic Data by Cell Type and Treatment
BJ
BMMSC
HCAEC
HEKn
HEMn
HSAEC
HSKM
HUVEC
HVSMC
NHA
NHO
PBMC
PreAdipo
WI38
PC1
PC2
Treatment
CTIS
EV
IRIS
OIS
OSIS
P

B

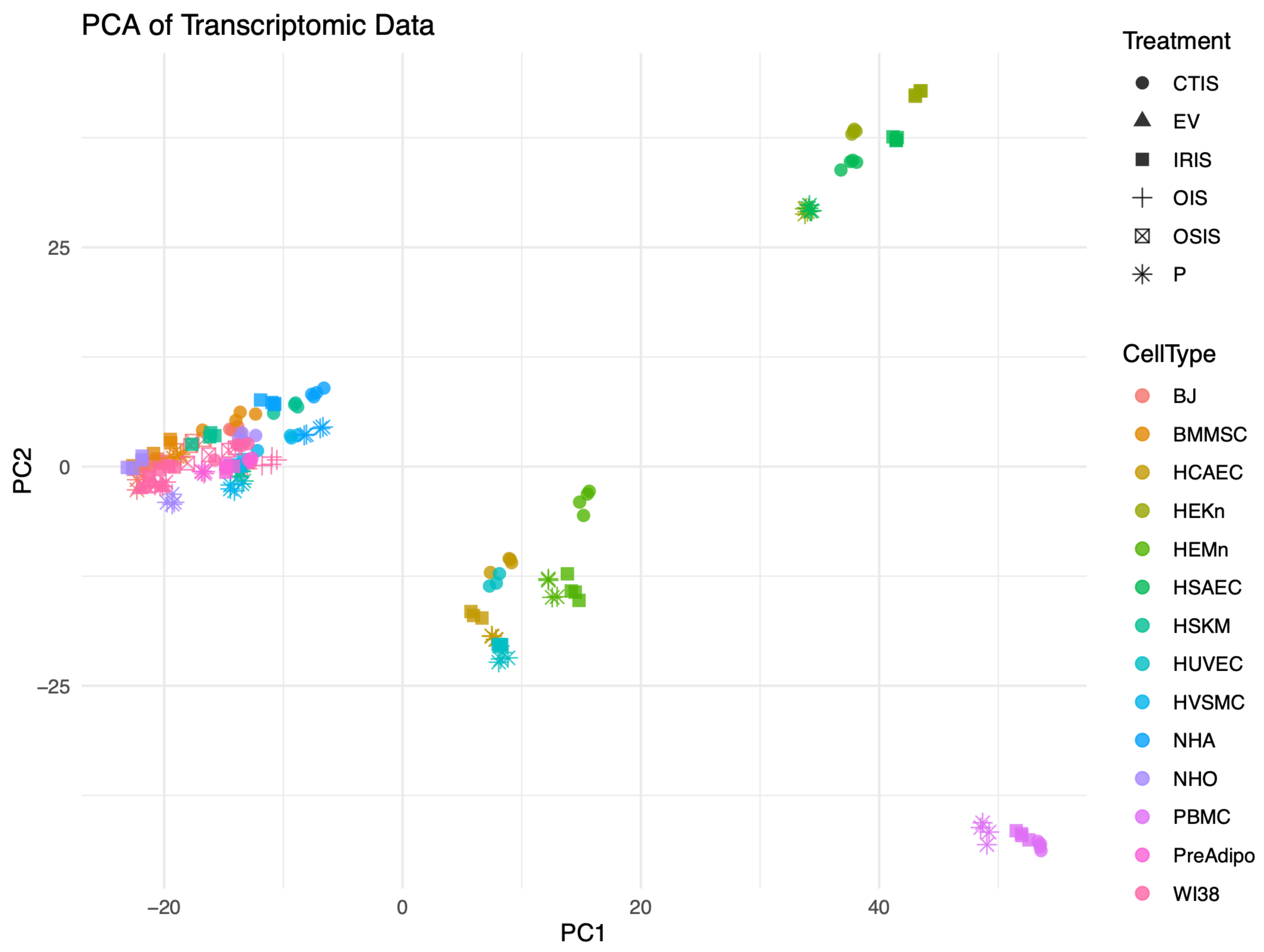
PCA of Transcriptomic Data
PC1
PC2
Treatment
CTIS
EV
IRIS
OIS
OSIS
P
CellType
BJ
BMMSC
HCAEC
HEKn
HEMn
HSAEC
HSKM
HUVEC
HVSMC
NHA
NHO
PBMC
PreAdipo
WI38

A

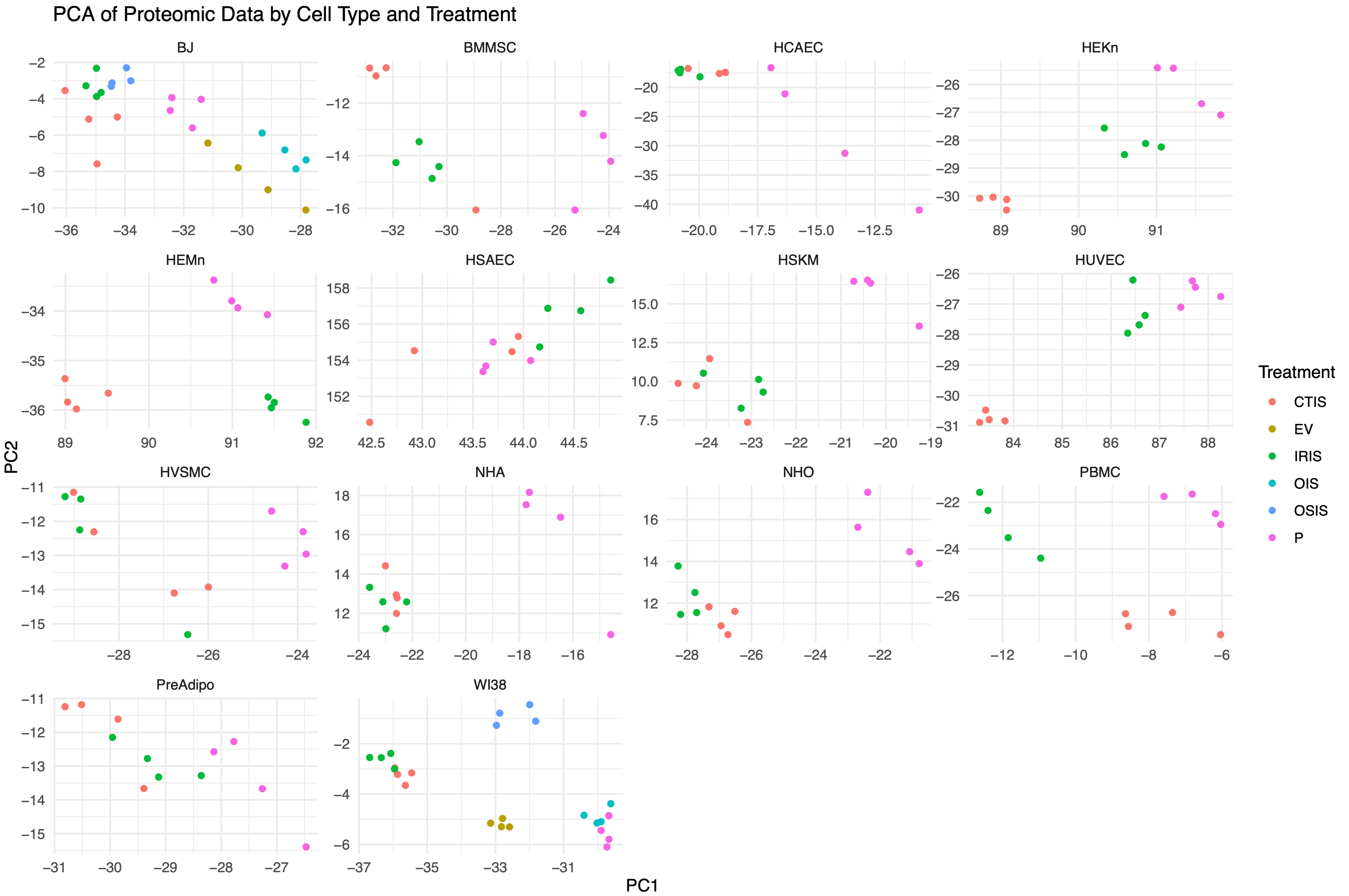
PCA of Proteomic Data by Cell Type and Treatment
BJ
BMMSC
HCAEC
HEKn
HEMn
HSAEC
HSKM
HUVEC
HVSMC
NHA
NHO
PBMC
PreAdipo
WI38
PC2
PC1
Treatment
CTIS
EV
IRIS
OIS
OSIS
P

B

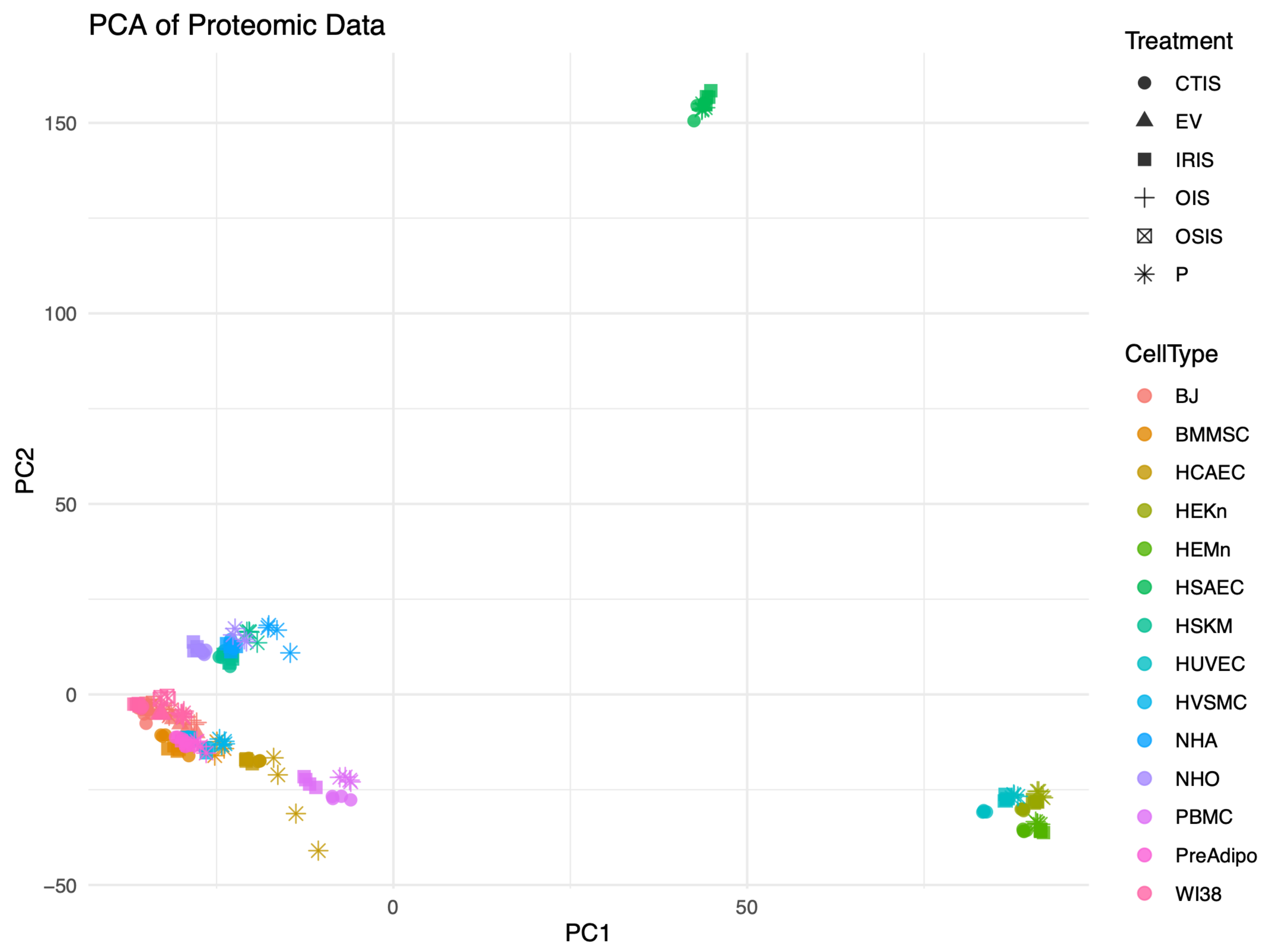
PCA of Proteomic Data
PC2
PC1
Treatment
CTIS
EV
IRIS
OIS
OSIS
P
CellType
BJ
BMMSC
HCAEC
HEKn
HEMn
HSAEC
HSKM
HUVEC
HVSMC
NHA
NHO
PBMC
PreAdipo
WI38

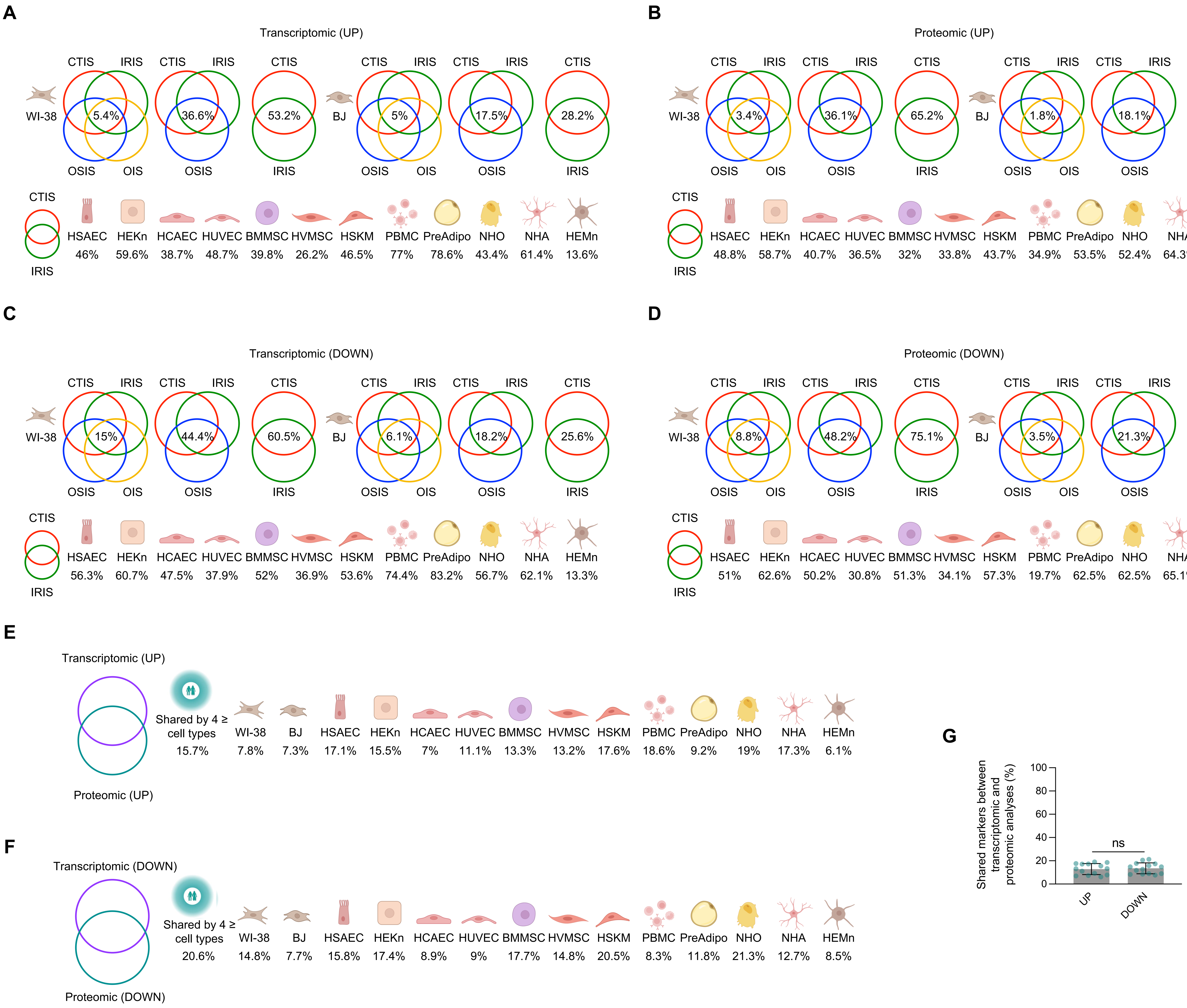
A
Transcriptomic (UP)
CTIS IRIS CTIS IRIS CTIS CTIS IRIS CTIS IRIS CTIS
WI-38 5.4% 36.6% 53.2% BJ 5% 17.5% 28.2%
OSIS OIS OSIS IRIS OSIS OIS OSIS IRIS
CTIS
IRIS
HSAEC HEKn HCAEC HUVEC BMMSC HVMSC HSKM PBMC PreAdipo NHO NHA HEMn
46% 59.6% 38.7% 48.7% 39.8% 26.2% 46.5% 77% 78.6% 43.4% 61.4% 13.6%
B
Proteomic (UP)
CTIS IRIS CTIS IRIS CTIS CTIS IRIS CTIS IRIS CTIS
WI-38 3.4% 36.1% 65.2% BJ 1.8% 18.1% 26.2%
OSIS OIS OSIS IRIS OSIS OIS OSIS IRIS
CTIS
IRIS
HSAEC HEKn HCAEC HUVEC BMMSC HVMSC HSKM PBMC PreAdipo NHO NHA HEMn
48.8% 58.7% 40.7% 36.5% 32% 33.8% 43.7% 34.9% 53.5% 52.4% 64.3% 14.5%
C
Transcriptomic (DOWN)
CTIS IRIS CTIS IRIS CTIS CTIS IRIS CTIS IRIS CTIS
WI-38 15% 44.4% 60.5% BJ 6.1% 18.2% 25.6%
OSIS OIS OSIS IRIS OSIS OIS OSIS IRIS
CTIS
IRIS
HSAEC HEKn HCAEC HUVEC BMMSC HVMSC HSKM PBMC PreAdipo NHO NHA HEMn
56.3% 60.7% 47.5% 37.9% 52% 36.9% 53.6% 74.4% 83.2% 56.7% 62.1% 13.3%
D
Proteomic (DOWN)
CTIS IRIS CTIS IRIS CTIS CTIS IRIS CTIS IRIS CTIS
WI-38 8.8% 48.2% 75.1% BJ 3.5% 21.3% 33.3%
OSIS OIS OSIS IRIS OSIS OIS OSIS IRIS
CTIS
IRIS
HSAEC HEKn HCAEC HUVEC BMMSC HVMSC HSKM PBMC PreAdipo NHO NHA HEMn
51% 62.6% 50.2% 30.8% 51.3% 34.1% 57.3% 19.7% 62.5% 62.5% 65.1% 8.5%
E
Transcriptomic (UP)
Proteomic (UP)
Shared by 4 ≥ cell types
15.7%
WI-38 BJ HSAEC HEKn HCAEC HUVEC BMMSC HVMSC HSKM PBMC PreAdipo NHO NHA HEMn
7.8% 7.3% 17.1% 15.5% 7% 11.1% 13.3% 13.2% 17.6% 18.6% 9.2% 19% 17.3% 6.1%
F
Transcriptomic (DOWN)
Proteomic (DOWN)
Shared by 4 ≥ cell types
20.6%
WI-38 BJ HSAEC HEKn HCAEC HUVEC BMMSC HVMSC HSKM PBMC PreAdipo NHO NHA HEMn
14.8% 7.7% 15.8% 17.4% 8.9% 9% 17.7% 14.8% 20.5% 8.3% 11.8% 21.3% 12.7% 8.5%
G
Shared markers between transcriptomic and proteomic analyses (%)
100
80
60
40
20
0
ns
UP
DOWN

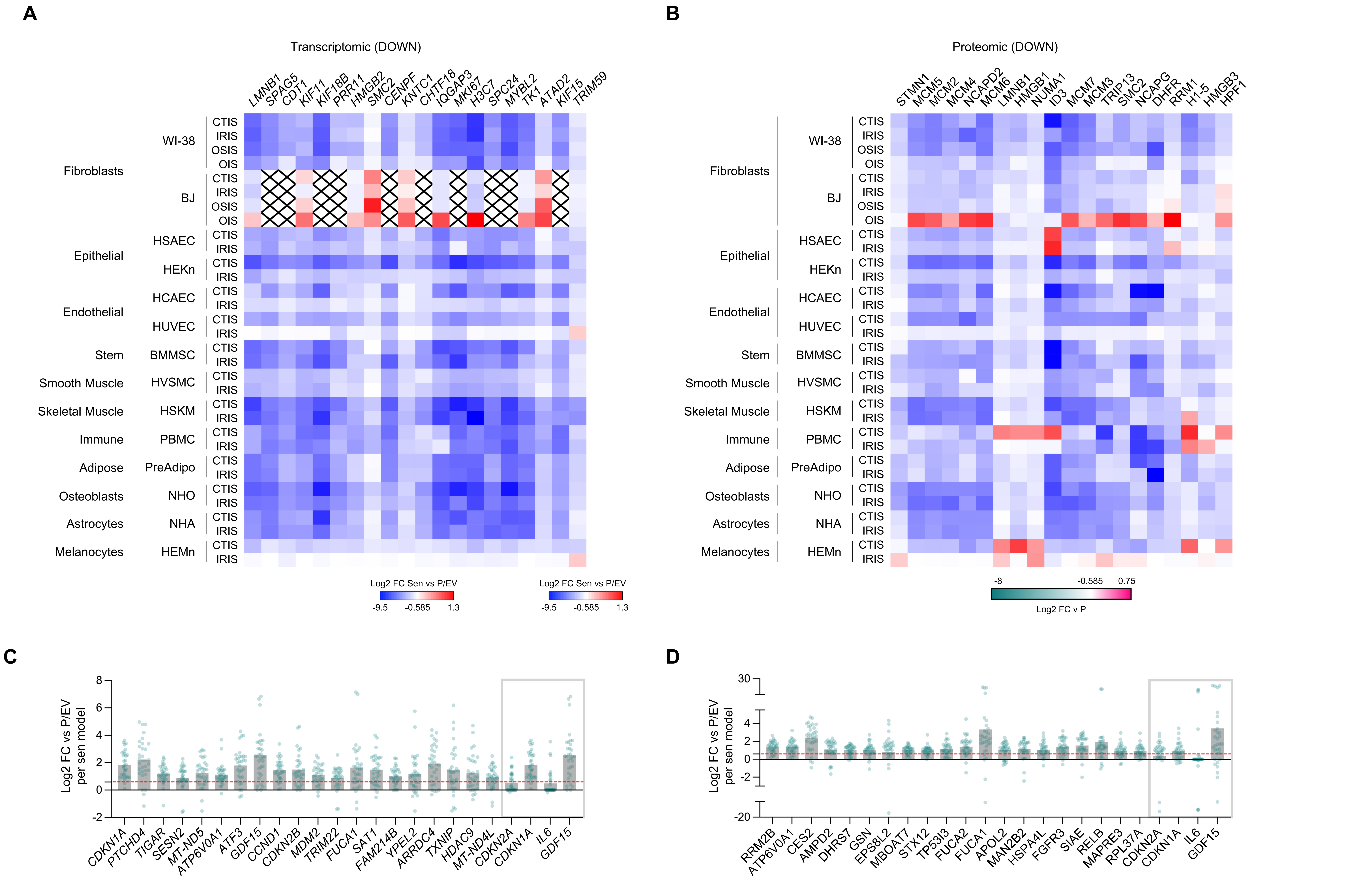
A
Transcriptomic (DOWN)
LMNB1 SPAG5 CDT1 KIF11 KIF18B PRR11 HMGB2 SMC2 CENPF KNTC1 CHTF18 IQGAP3 MKI67 H3C7 SPC24 MYBL2 TK1 ATAD2 KIF15 TRIM59
Fibroblasts
WI-38
BJ
CTIS
IRIS
OSIS
OIS
Epithelial
HSAEC
HEKn
Endothelial
HCAEC
HUVEC
Stem
BMMSC
Smooth Muscle
HVSMC
Skeletal Muscle
HSKM
Immune
PBMC
Adipose
PreAdipo
Osteoblasts
NHO
Astrocytes
NHA
Melanocytes
HEMn
Log2 FC Sen vs P/EV
-9.5 -0.585 1.3
Log2 FC Sen vs P/EV
-9.5 -0.585 1.3
B
Proteomic (DOWN)
STMN1 MCM5 MCM2 MCM4 NCAPD2 MCM6 LMNB1 HMGB1 NUMA1 ID3 MCM7 MCM3 TRIP13 SMC2 NCAPG DHFR RRM1 H1-5 HMGB3 HPF1
-8 -0.585 0.75
Log2 FC v P
C
Log2 FC vs P/EV per sen model
8
6
4
2
0
-2
CDKN1A PTCHD4 TIGAR SESN2 MT-ND5 ATP6V0A1 ATF3 GDF15 CCND1 CDKN2B MDM2 TRIM22 FUCA1 SAT1 FAM214B YPEL2 ARRDC4 TXNIP HDAC9 MT-ND4L CDKN2A CDKN1A IL6 GDF15
D
Log2 FC vs P/EV per sen model
30
4
2
0
-2
-20
RRM2B ATP6V0A1 CES2 AMPD2 DHRS7 GSN EPS8L2 MBOAT7 STX12 TP53I3 FUCA2 FUCA1 APOL2 MAN2B2 HSPA4L FGFR3 SIAE RELB MAPRE3 RPL37A CDKN2A CDKN1A IL6 GDF15

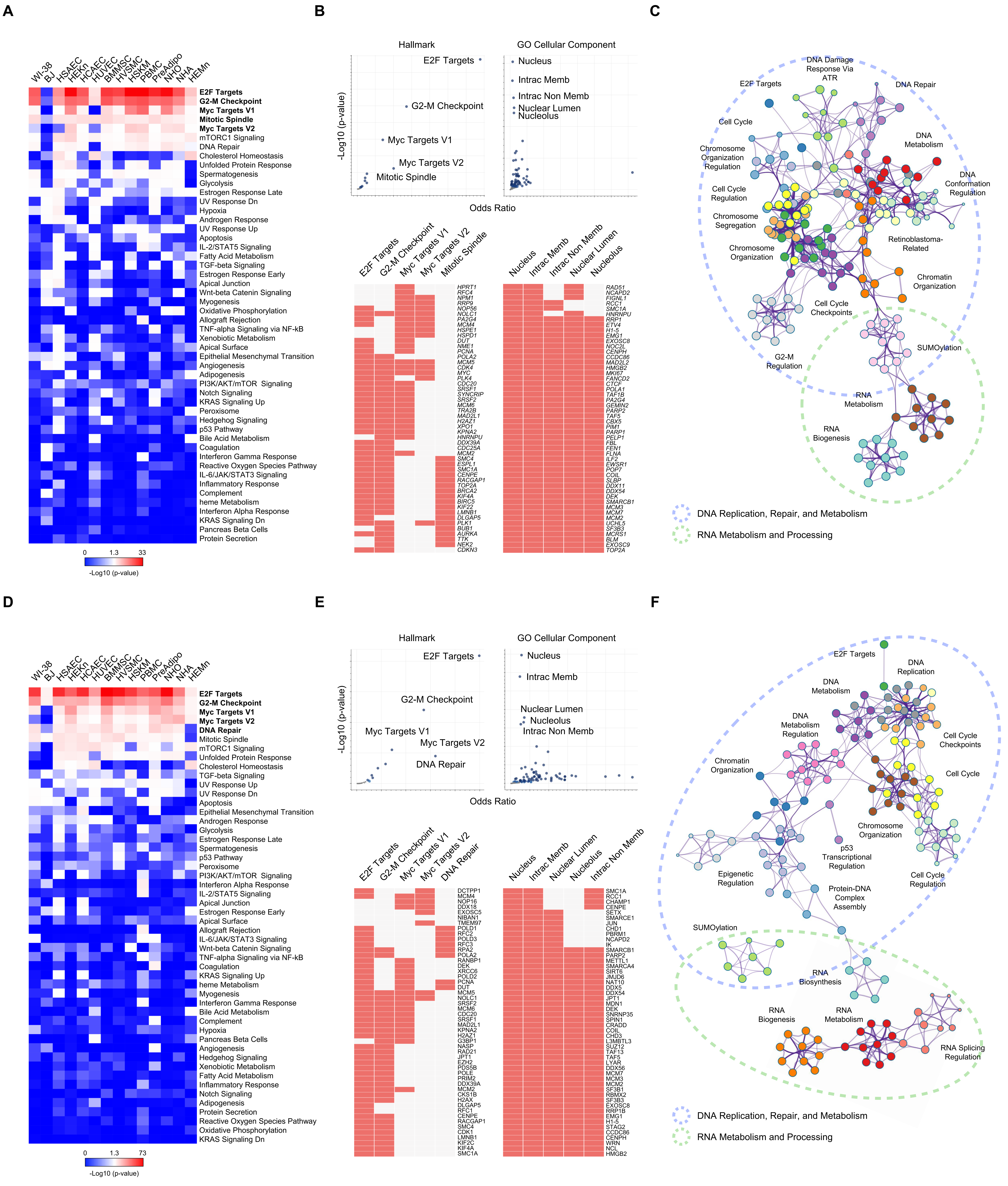
A
WI-38
BJ
HSAEC
HEKn
HCAEC
HUVEC
BMMSC
HVSMC
HSKM
PBMC
PreAdipo
NHO
NHA
HEMn
E2F Targets
G2-M Checkpoint
Myc Targets V1
Mitotic Spindle
Myc Targets V2
mTORC1 Signaling
DNA Repair
Cholesterol Homeostasis
Unfolded Protein Response
Spermatogenesis
Glycolysis
Estrogen Response Late
UV Response Dn
Hypoxia
Androgen Response
UV Response Up
Apoptosis
IL-2/STAT5 Signaling
Fatty Acid Metabolism
TGF-beta Signaling
Estrogen Response Early
Apical Junction
Wnt-beta Catenin Signaling
Myogenesis
Oxidative Phosphorylation
Allograft Rejection
TNF-alpha Signaling via NF-kB
Xenobiotic Metabolism
Apical Surface
Epithelial Mesenchymal Transition
Angiogenesis
Adipogenesis
PI3K/AKT/mTOR Signaling
Notch Signaling
KRAS Signaling Up
Peroxisome
Hedgehog Signaling
p53 Pathway
Bile Acid Metabolism
Coagulation
Interferon Gamma Response
Reactive Oxygen Species Pathway
IL-6/JAK/STAT3 Signaling
Inflammatory Response
Complement
heme Metabolism
Interferon Alpha Response
KRAS Signaling Dn
Pancreas Beta Cells
Protein Secretion
0
1.3
33
-Log10 (p-value)
B
Hallmark
GO Cellular Component
-Log10 (p-value)
Odds Ratio
E2F Targets
G2-M Checkpoint
Myc Targets V1
Myc Targets V2
Mitotic Spindle
Nucleus
Intrac Memb
Intrac Non Memb
Nuclear Lumen
Nucleolus
C
DNA Damage Response Via ATR
E2F Targets
DNA Repair
Cell Cycle
DNA Metabolism
Chromosome Organization Regulation
DNA Conformation Regulation
Cell Cycle Regulation
Chromosome Segregation
Retinoblastoma-Related
Chromosome Organization
Chromatin Organization
Cell Cycle Checkpoints
SUMOylation
G2-M Regulation
RNA Metabolism
RNA Biogenesis
DNA Replication, Repair, and Metabolism
RNA Metabolism and Processing
D
WI-38
BJ
HSAEC
HEKn
HCAEC
HUVEC
BMMSC
HVSMC
HSKM
PBMC
PreAdipo
NHO
NHA
HEMn
E2F Targets
G2-M Checkpoint
Myc Targets V1
Myc Targets V2
DNA Repair
Mitotic Spindle
mTORC1 Signaling
Unfolded Protein Response
Cholesterol Homeostasis
TGF-beta Signaling
UV Response Up
UV Response Dn
Apoptosis
Epithelial Mesenchymal Transition
Androgen Response
Glycolysis
Estrogen Response Late
Spermatogenesis
p53 Pathway
Peroxisome
PI3K/AKT/mTOR Signaling
Interferon Alpha Response
IL-2/STAT5 Signaling
Apical Junction
Estrogen Response Early
Apical Surface
Allograft Rejection
IL-6/JAK/STAT3 Signaling
Wnt-beta Catenin Signaling
TNF-alpha Signaling via NF-kB
Coagulation
KRAS Signaling Up
heme Metabolism
Myogenesis
Interferon Gamma Response
Bile Acid Metabolism
Complement
Hypoxia
Pancreas Beta Cells
Angiogenesis
Hedgehog Signaling
Xenobiotic Metabolism
Fatty Acid Metabolism
Inflammatory Response
Notch Signaling
Adipogenesis
Protein Secretion
Reactive Oxygen Species Pathway
Oxidative Phosphorylation
KRAS Signaling Dn
0
1.3
73
-Log10 (p-value)
E
Hallmark
GO Cellular Component
-Log10 (p-value)
Odds Ratio
E2F Targets
G2-M Checkpoint
Myc Targets V1
Myc Targets V2
DNA Repair
Nucleus
Intrac Memb
Nuclear Lumen
Nucleolus
Intrac Non Memb
F
E2F Targets
DNA Replication
DNA Metabolism
DNA Metabolism Regulation
Cell Cycle Checkpoints
Chromatin Organization
Cell Cycle
Chromosome Organization
p53 Transcriptional Regulation
Epigenetic Regulation
Cell Cycle Regulation
Protein-DNA Complex Assembly
SUMOylation
RNA Biosynthesis
RNA Biogenesis
RNA Metabolism
RNA Splicing Regulation
DNA Replication, Repair, and Metabolism
RNA Metabolism and Processing

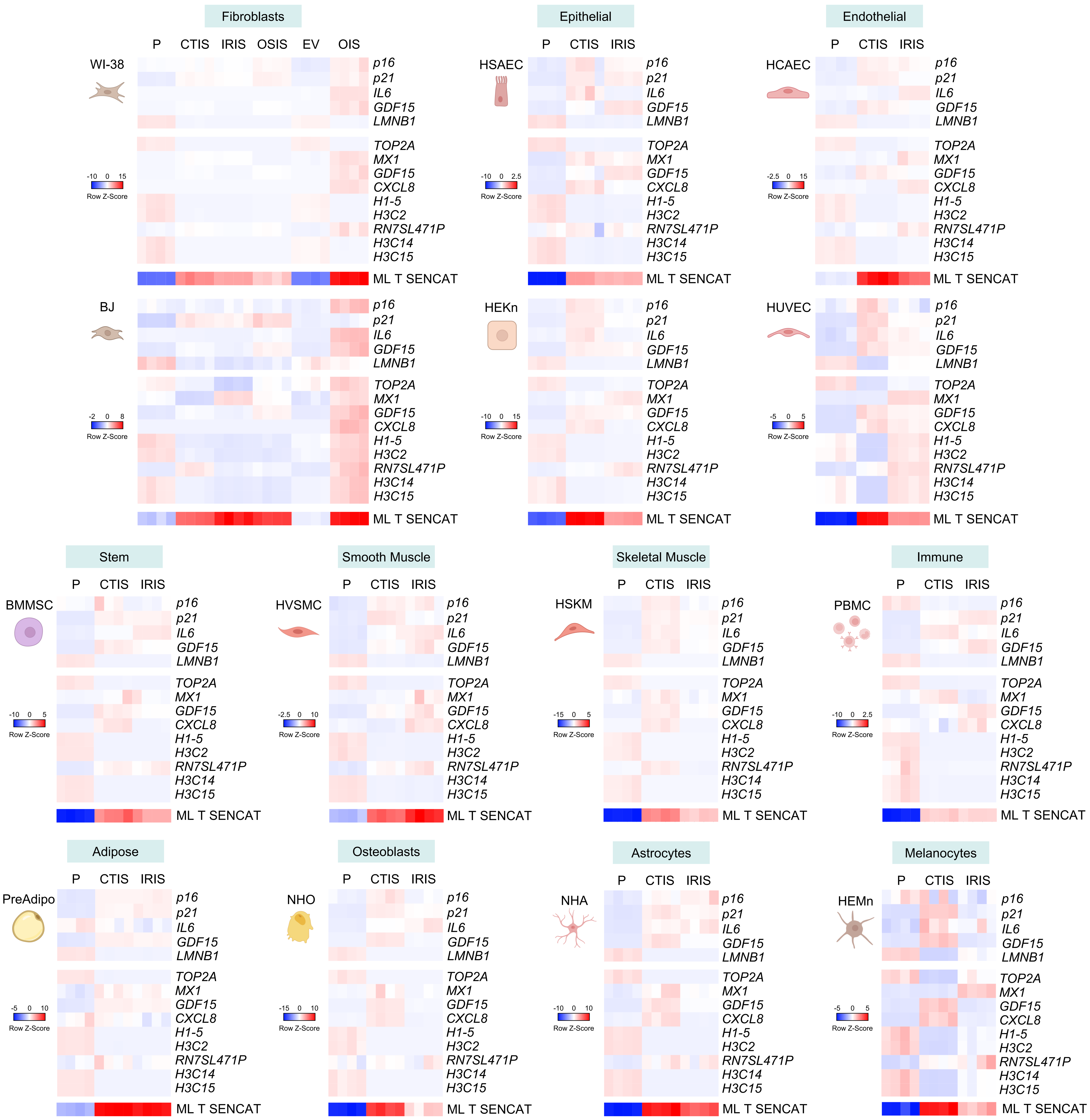
Fibroblasts
Epithelial
Endothelial
P CTIS IRIS OSIS EV OIS
P CTIS IRIS
WI-38
BJ
HSAEC
HEKn
HCAEC
HUVEC
p16
p21
IL6
GDF15
LMNB1
TOP2A
MX1
CXCL8
H1-5
H3C2
RN7SL471P
H3C14
H3C15
ML T SENCAT
Row Z-Score
Stem
Smooth Muscle
Skeletal Muscle
Immune
BMMSC
HVSMC
HSKM
PBMC
Adipose
Osteoblasts
Astrocytes
Melanocytes
PreAdipo
NHO
NHA
HEMn

Anerillas et al. Figure S10.

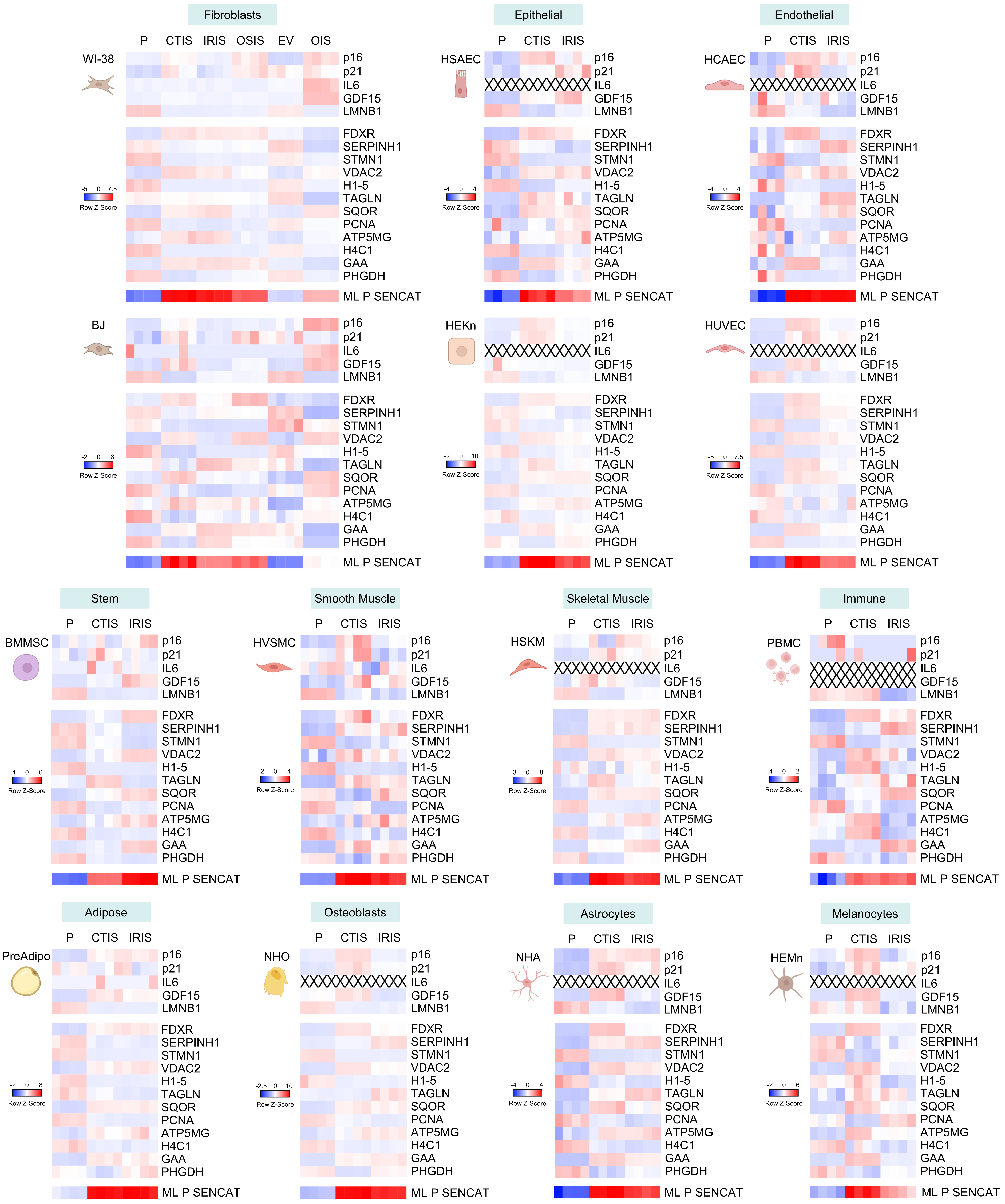

A

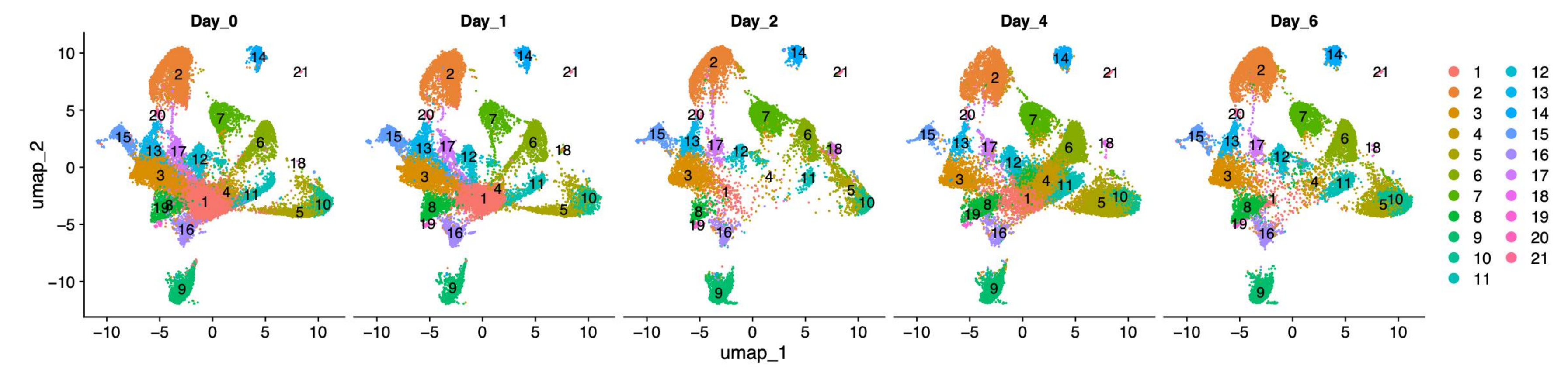

B

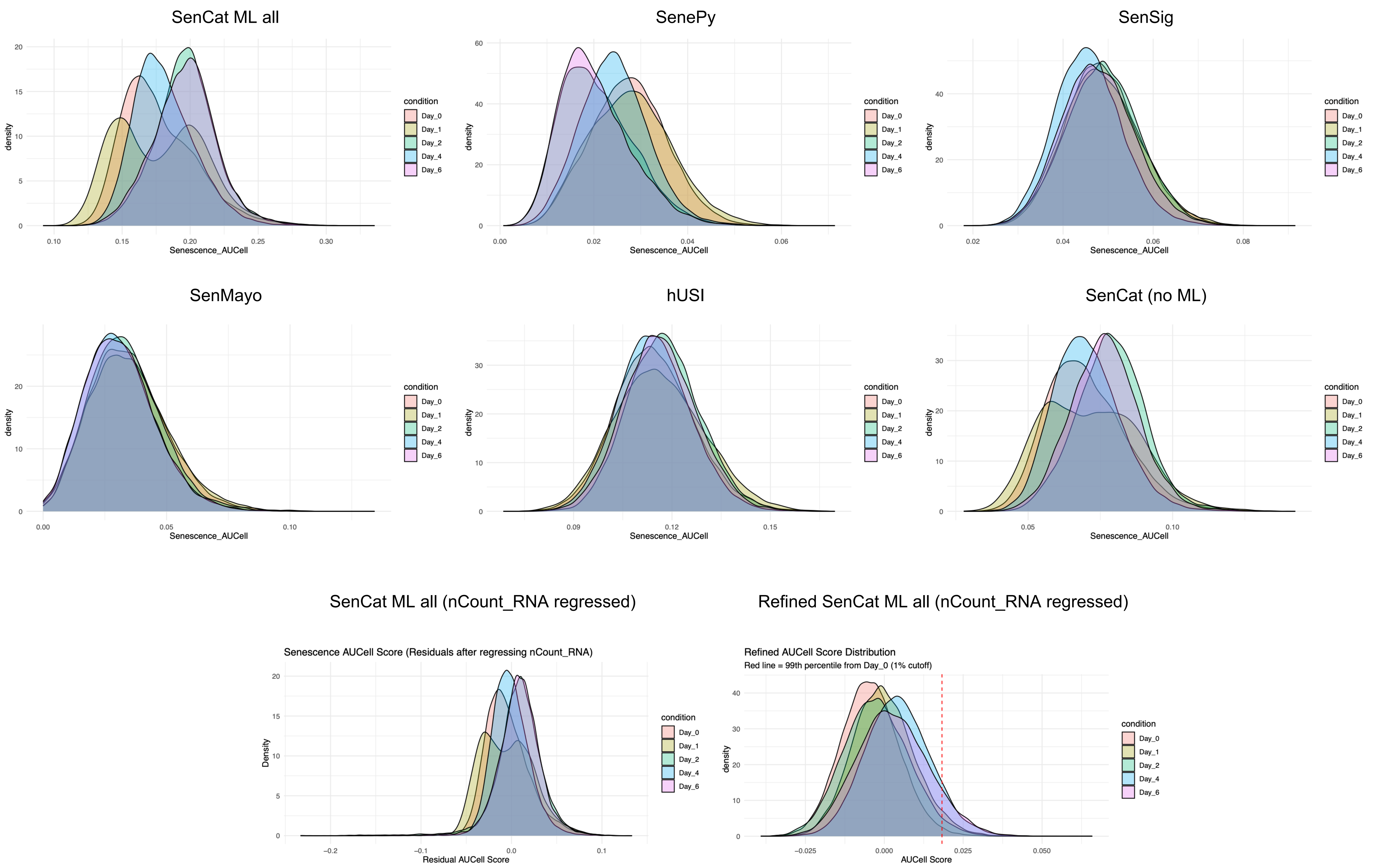

C

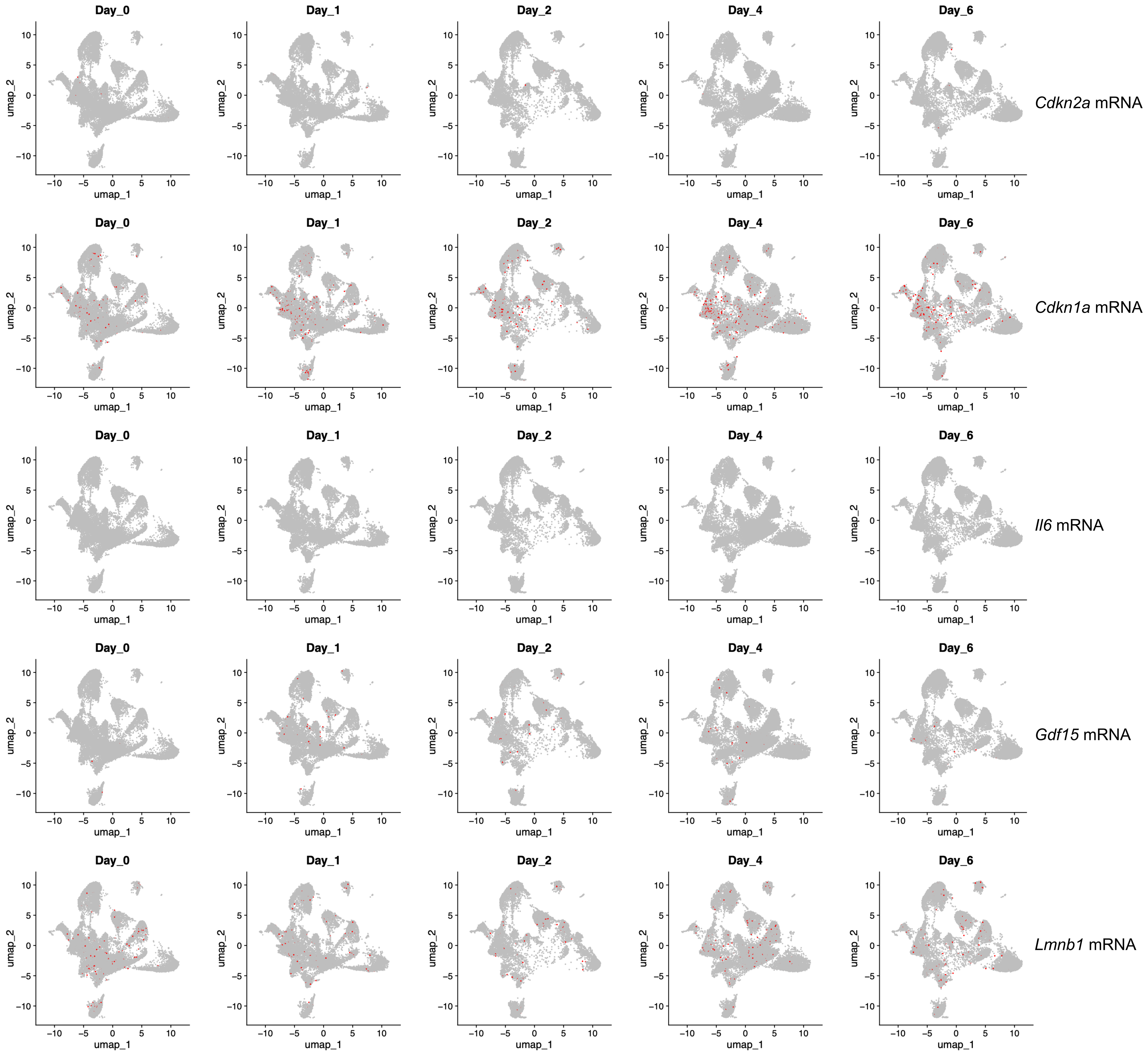

D

Condition	Total cells	Senescent cells	Percentage senescent
Day_0	30783	308	1
Day_1	19916	775	3.89
Day_2	13448	374	2.78
Day_4	29505	2646	8.97
Day_6	16716	1517	9.08

A

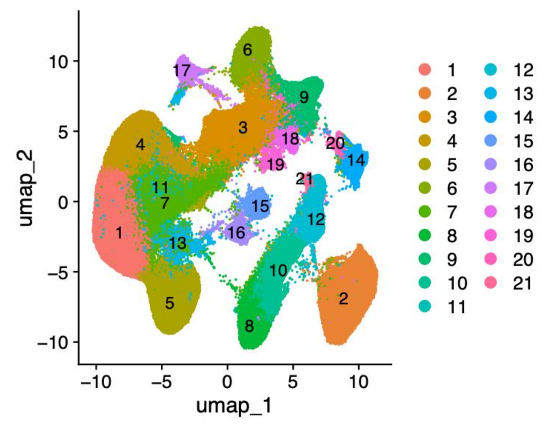

B

Cluster	Cell Type
1	Epithelial (Proximal Tubule)
2	Epithelial (Distal Convoluted Tube)
3	Epithelial (Proximal Tubule)
4	Epithelial (Proximal Tubule)
5	Epithelial (Proximal Tubule)
6	Epithelial (Proximal Tubule)
7	Epithelial (Proximal Tubule)
8	Epithelial (Distal Convoluted Tube)
9	Podocyte
10	Epithelial (Distal Convoluted Tube)
11	Epithelial (Proximal Tubule)
12	Epithelial (Collecting Duct)
13	Epithelial (Proximal Tubule)
14	Epithelial (Collecting Duct)
15	Epithelial (Collecting Duct)
16	Epithelial (Collecting Duct)
17	Podocyte
18	Smooth Muscle Cell
19	Macrophages
20	Fibroblast
21	Epithelial (Distal Convoluted Tube)

C

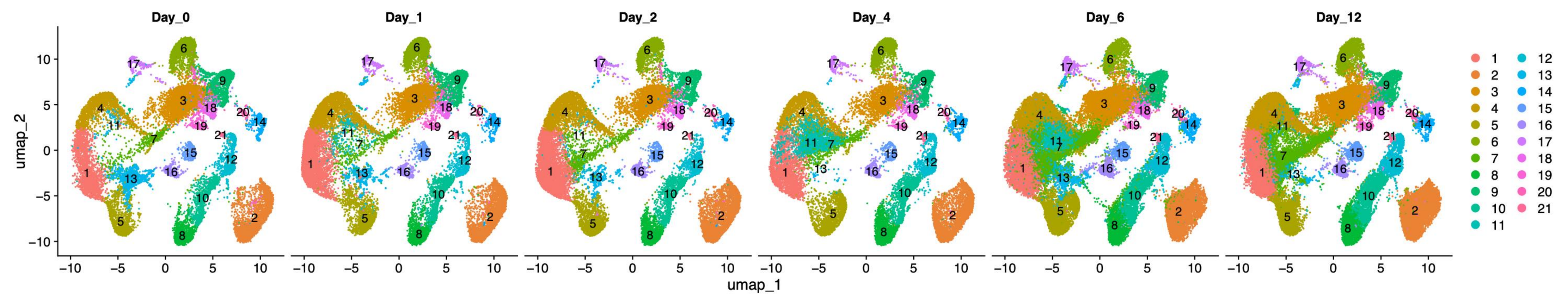

D

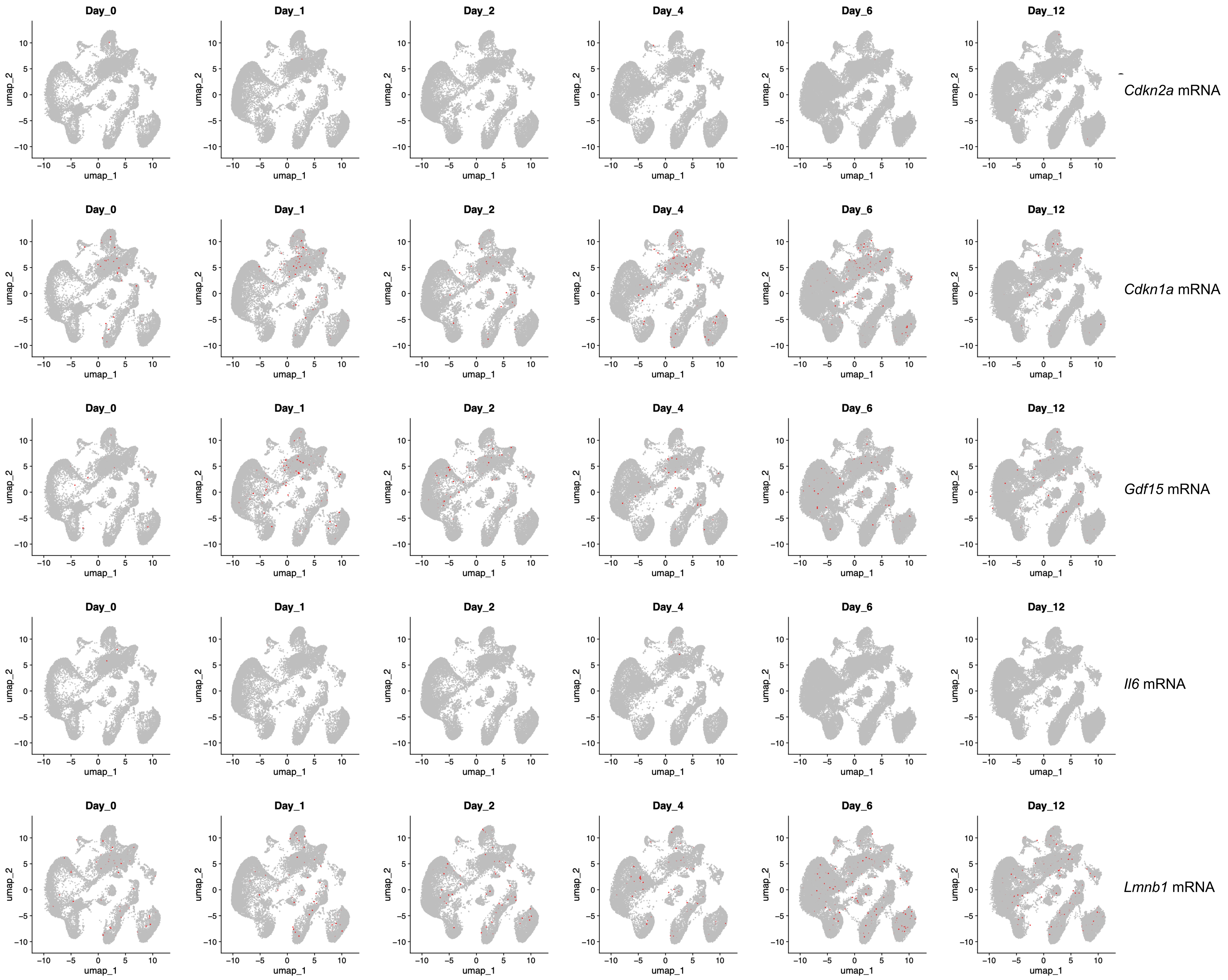

E

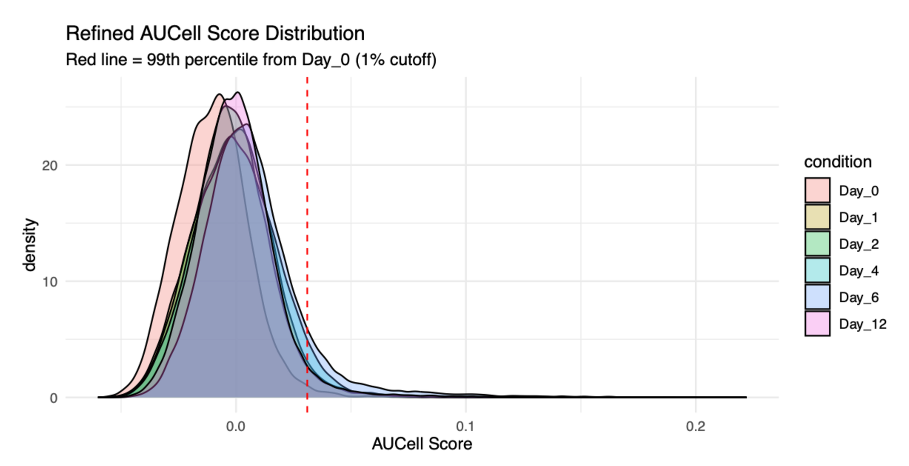

F

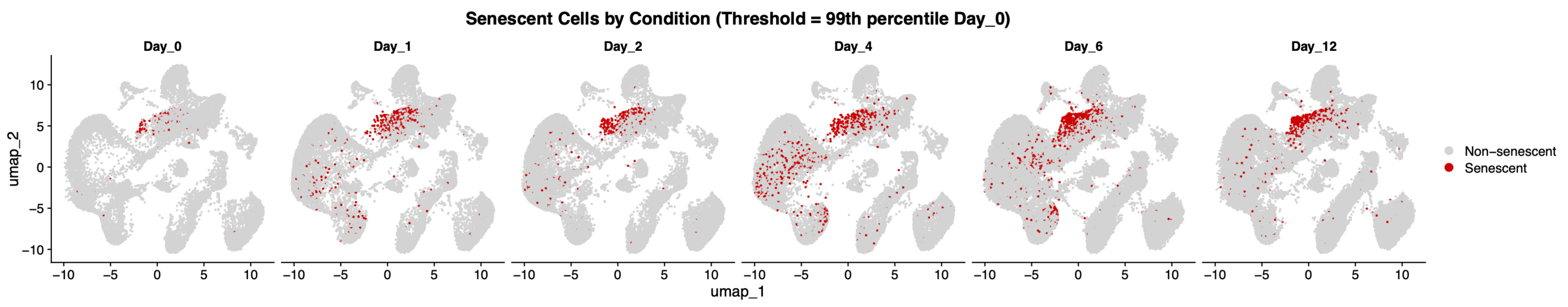

G

condition	cells	senescent_cells	percent_senescent
Day_0	22049	221	1
Day_1	25351	863	3.4
Day_2	22582	716	3.17
Day_4	21961	1083	4.93
Day_6	43794	4292	9.8
Day_12	49446	1881	3.8

H

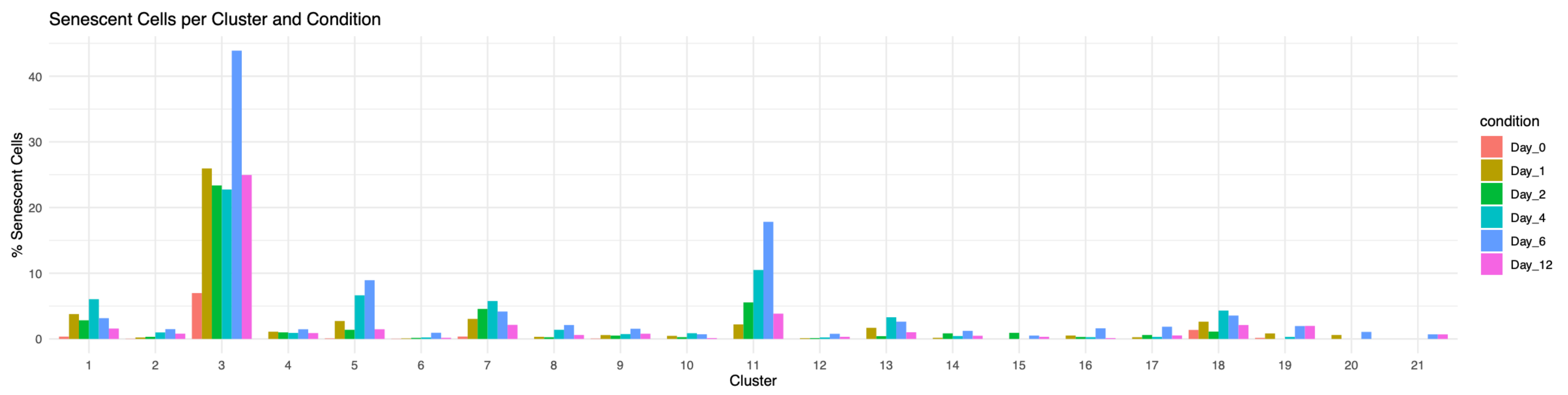

A

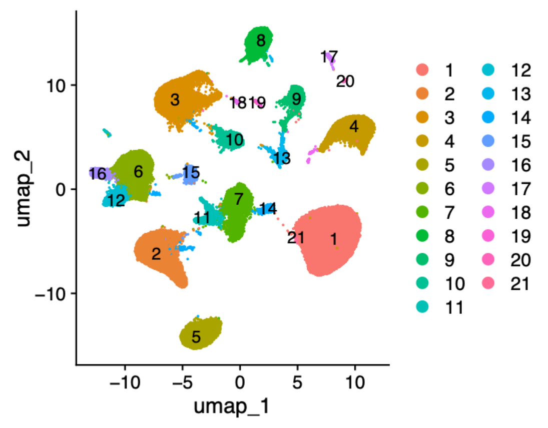

B

Cluster	Cell type
1	Epithelial (AT2)
2	B Cell
3	Endothelial
4	Epithelial (Clara cell)
5	Macrophage
6	T Cell
7	Monocyte
8	Epithelial (AT1)
9	Smooth Muscle Cell
10	Endothelial
11	Monocyte
12	T Cell
13	Fibroblast
14	Neutrophil
15	B Cell
16	T Cell
17	Epithelial (Ciliated cell)
18	Fibroblast
19	Epithelial (AT1)
20	Fibroblast
21	Epithelial (AT2)

C

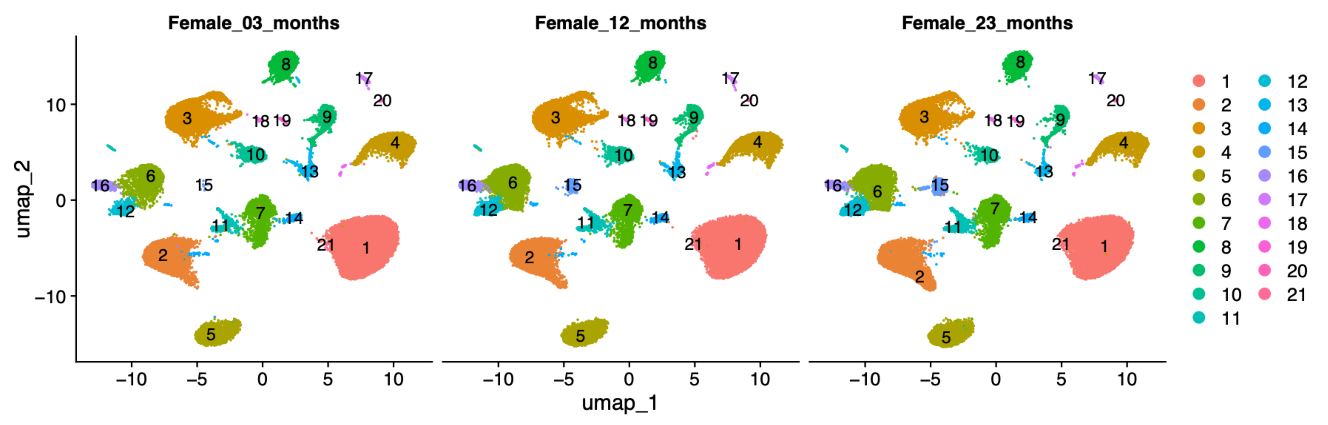

D

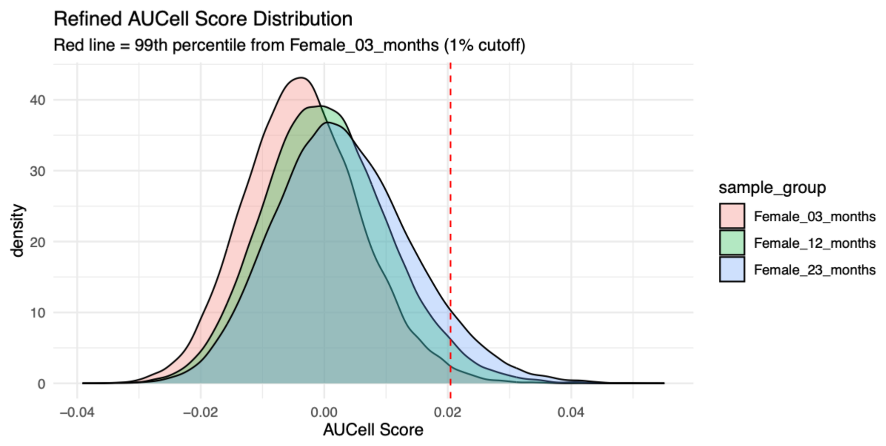

E

sample_gro	total_cells	senescent_cells	percent_senescent
Female_03_	40375	404	1
Female_12_	40212	1301	3.24
Female_23_	36849	2572	6.98

F

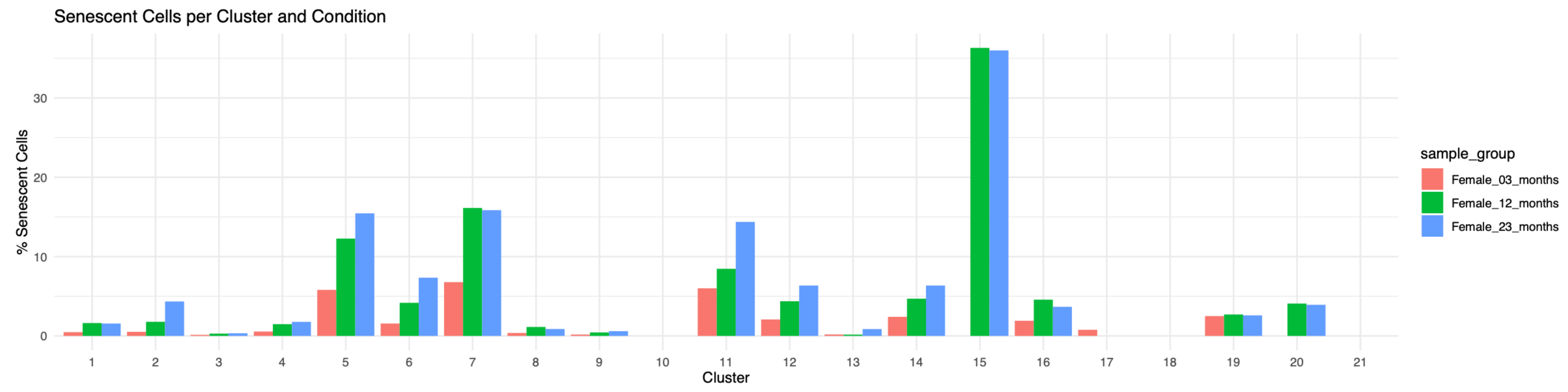

G

interacting_pair	gene_a	gene_b	Senescent|Neighbor
FGF10_FGFR2	FGF10	FGFR2	1,502
EFNA5_EPHA7	EFNA5	EPHA7	713
SEMA3A_NRP1	SEMA3A	NRP1	604
SEMA3A_PlexinA4_complex1	SEMA3A		503
IGF1_IGF1R	IGF1	IGF1R	471
SEMA6A_PlexinA2_complex1	SEMA6A		410
THBS1_CD36	THBS1	CD36	396
TNC_integrin_a9b1_complex	TNC		378
NRXN1_DAG1	NRXN1	DAG1	285
TGFB2_TGFbeta_receptor2	TGFB2		202
